# Predictive efficacy of the preoperative neutrophil–lymphocyte ratio in lymph node metastasis of cN0 hormone receptor-positive breast cancer

**DOI:** 10.1038/s41598-024-63318-y

**Published:** 2024-06-20

**Authors:** Miao-Feng Wang, Jia-Rui Cai, Heng Xia, Xiu-feng Chu

**Affiliations:** Department of Thyroid and Breast Surgery, Shaoxing Central Hospital (The Central Hospital of Shaoxing University), Shaoxing, 312030 Zhejiang Province China

**Keywords:** Breast cancer, Neutrophil-to-lymphocyte ratio, Clinical stage, Lymph node metastasis, Cancer, Cell biology, Immunology

## Abstract

Breast cancer, as the most common cancer, has surpassed lung cancer worldwide. The neutrophil-to-lymphocyte ratio (NLR) has been linked to the onset of cancer and its prognosis in recent studies. However, quite a few studies have shown that there is a link between NLR and lymph node metastases in cN0 hormone receptor-positive (HR(+)) breast cancer. The purpose of this study was to evaluate the correlation between NLR and lymph node metastases in cN0 HR(+) breast cancer patients. From January 2012 to January 2022, 220 patients with cN0 HR(+) invasive breast cancers were enrolled in this study. The relationship between NLR and pathological data was statistically examined. The receiver operating characteristic (ROC) curve was used to determine the optimal cutoff of NLR, a chi-squared test was used for the univariate analysis, and logistic analysis was used for the multivariate analysis. The NLR had an optimal cutoff of 2.4 when the Jorden index was at a maximum. Patients with axillary lymph node metastases had a higher NLR (*P* < 0.05). A Univariate analysis showed that there were significant differences in cN0 HR(+) breast cancer with axillary lymph node metastasis among different clinical stages, histological grades, Ki-67 levels, tumor sizes, and NLR levels (*P* < 0.05). Clinical stage, tumor size, and NLR were found to be independent risk factors for lymph node metastases in multifactorial analysis. In cN0 HR(+) breast cancer, NLR is an independent risk factor for lymph node metastases. An NLR ≥ 2.4 indicates an increased probability of lymph node metastases. An elevated preoperative NLR has a high predictive value for axillary lymph node metastases.

## Introduction

Breast cancer is the most common malignant tumor in women, of which early-stage (stage I–II) breast cancer (EBC) accounts for 73.1% in China^1,2^. It is widely known that clinical stages, lymph node metastasis, histological grade, Ki-67 levels, ER, PR, human epidermal growth factor receptor 2 (HER-2) status, and molecular typing are used for the determination of postoperative adjuvant treatment. However, the aforementioned indicators can only be acquired via surgery. Axillary lymph node metastasis is one of the main significant factors of prognostic indicators in breast cancer^3^. Nevertheless, due to a lack of early diagnostic biomarker indicators for lymph node metastases, a proportion of patients underwent unecessary axillary lymph node dissection (ALND). Therefore, finding an effective and less invasive biomarker indicator for predicting lymph node metastases to prevent the complications of unnecessary ALND is imperative.

Inflammation has been identified as one of the six biological characteristics of tumor formation and a hallmark of cancer since its discovery^4^. Chronic inflammation has recently been discovered to play an important role in tumor formation, progression, invasion, and metastasis^5^. Rudolf Virchow was the first to notice the link between inflammation and cancer when he discovered leukocytes within tumors and hypothesized that inflammation increased cellular proliferation^6,7^. The neutrophil-to-lymphocyte ratio (NLR) is a novel assay that is inexpensive, simple, repeatable, and easy to measure^8,9^. An increased NLR has been reported to be an independent risk factor for poor prognosis in malignancies of the lungs, esophagus, pancreas, and head and neck^10–13^. Because of its accessibility and ease of calculation from patients' routine blood cell counts, the NLR has been proposed as a promising marker of cancer prognosis.

The relationship between neutrophils and cancer prognosis has been studied in numerous systematic reviews and meta-analyses. However, there has been limited research on NLR and its ability to predict lymph node metastases in HR(+) breast cancer. We therefore investigates the relationship between clinicopathological features and lymph node metastases in this study. We further studied the predictive efficacy of NLR for cN0 HR(+) breast cancer lymph node metastasis.

## Patients and methods

A total of 220 cN0 hormone receptor-positive breast cancer (HR(+)) breast cancer patients who underwent sentinel lymph node biopsy (SLNB) or ALND at the Shaoxing Central Hospital between January 2012 and January 2022 were included in this study. Of these patients, 182 underwent SLNB, while the remaining 38 patients directly proceeded to axillary lymph node dissection based on clinical judgment and patient preference. Age, tumor size, ER, PR, HER-2, Ki67, histological grade, NLR, and lymph node metastases were all gathered as clinical data. Routine blood tests were obtained within one week before surgery. The Shaoxing Central Hospital's Institutional Review Committee approved this study, and waived off requirement for informed patient consent due to it being a retrospective study.All methods were performed in accordance with the relevant guidelines and regulations.

Inclusion criteria: individuals with cT1-2N0M0 HR(+) invasive breast cancer confirmed by histology. Exclusion criteria: HER-2 positive breast cancer; patients with latent infection or active infection, hematologic disorders, steroid treatment, diseases of the immune system or oral immunosuppressive; or a combination of other malignant tumors.

The NLR was calculated as the neutrophils divided by the lymphocytes, the calculation of NLR is made with the absolute count value of the parameters. The 8th edition of the American Joint Committee on Cancer (AJCC-8th) handbook was used to describe the TNM staging of BC^14^. The immunohistochemistry results were as follows: the cutoff value of estrogen receptor (ER) and progesterone receptor (PR) was set at 1%, < 1% was negative, and ≥ 1% was positive. The Ki-67 cutoff value was set at 20%, with < 20% indicating low expression and ≥ 20% indicating high expression^15^. According to the current ASCO/CAP recommendations, HER2 positivity is defined as a FISH ratio ≥ 2, a HER2 gene copy number (CN) of 6.0, or an immunohistochemistry (IHC) score of 3 + ^16^.

## Statistical analysis

SPSS 26.0 was used to analyze the data in this study. The NLR was calculated using the mean ± standard deviation (x ± sd) method. Patients were split into two groups based on the presence of lymph node metastases: positive and negative. The ROC curve was created. The maximal Youden index was used to calculate NLR cutoff values, and patients were divided into high and low groups. The chi-squared test was used to assess differences in the two groups with varying age, tumor size, ER, PR, Ki67, drinking, smoking, menopause, and lymph node metastases. Multivariate logistic regression was used for multivariate analysis. Statistical significance was defined as a *P* < 0.05.

## Results

**Figure1:**
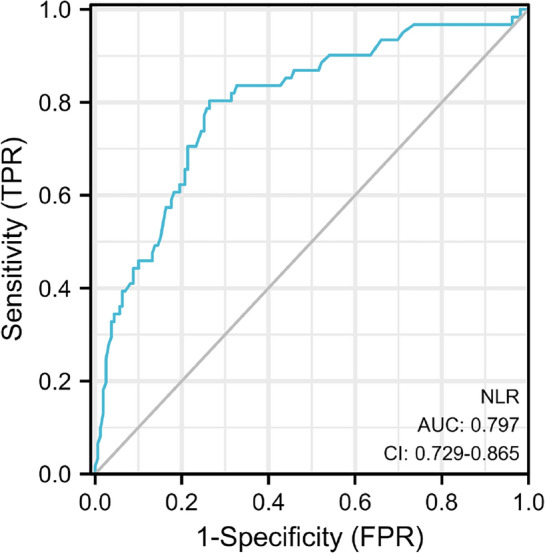
ROC curve of the relationship between preoperative NLR level and lymph node metastases. ROC: Receiver operating characteristic; NLR: neutrophil to lymphocyte ratio.

There were 220 patients in this research, with a median age of 55 years (range 31–76 years). Table 1 shows the baseline characteristics of the patients. The relationship between NLR and lymph node metastases was used to create ROC curves. The optimal cutoff value of NLR, according to the ROC curve and the Youden index, was 2.4. The estimated area under the curve is 0.797 (Fig. 1).

**Table 1:** Demographic data, clinicopathologic characteristics.

	Cases	Constituent ratio (%)
Menopause		
Yes	112	51
No	108	49
Tumor size (cm)		
> 2	109	49.5
≤ 2	111	50.5
Ki-67		
High expression	109	49.5
Low expression	111	50.5
Lymph node metastases		
Positive	61	27.8
Negative	159	72.2
NLR		
High	91	41.4
Low	129	58.6
Smoking		
Yes	5	2.3
No	215	97.7
Drinking		
Yes	16	7.3
No	204	92.7
Histologic grade		
G3	48	21.8
G1/2	172	78.2
Clinical stages		
III–IV	33	15
I–II	187	85

Patients were split into two groups based on the NLR cutoff value: the NLR value high level (≥ 2.4) group and NLR value low level (< 2.4) group. Significant differences were found between the two groups regarding different clinical stages, lymph node metastases, Ki-67, tumor sizes, and histologic grades, as shown in Table 2.

**Table 2:** The association between NLR level and clinicopathological characteristics.

	NLR		*P*
	High(91)	Low(129)	
Menopause			
Yes	40	72	0.083
No	51	57	
Tumor size (cm)			
> 2	69	40	< 0.0005
≤ 2	22	89	
Ki-67			
High expression	65	44	< 0.0005
Low expression	26	85	
Lymph node metastases			
Positive	49	12	< 0.0005
Negative	42	117	
Smoking			
Yes	2	3	0.692
No	89	126	
Drinking			
Yes	7	9	0.841
No	84	120	
Histologic grade			
G3	29	19	0.002
G1/2	62	110	
Clinical stages			
III–IV	22	11	0.001
I–II	69	118	

In this research, 61 individuals had lymph node metastases, accounting for 27.7% (61/220) of the total. The outcomes of the univariate analysis showed statistically significant variations in lymph node metastases between groups with varied tumor sizes, NLR levels, clinical stages, Ki-67 levels, and histologic grade (*P* < 0.05) (Table 3). A multivariate analysis showed that patients with clinical stages III–IV, tumor size > 2 cm, and NLR ≥ 2.4 had a higher rate of lymph node metastasis than individuals with clinical stages I–II, tumor size < 2 cm, and NLR < 2.4 (Table 4). Patients with NLR ≥ 2.4 had a higher probability of lymph node metastases than patients with NLR < 2.4.

**Table 3:** The association between lymph node metastasis and clinicopathological features (Univariate analysis).

	lymph node metastases		X2	*P*
	Yes(61)	No(159)		
Menopause				
Yes	25	87	3.327	0.068
No	36	72		
Tumor size (cm)				
> 2	39	70	6.990	0.008
≤ 2	22	89		
Ki-67				
High expression	41	68	10.539	0.001
Low expression	20	91		
NLR				
High	49	42	52.832	< 0.0005
Low	12	117		
Smoking				
Yes	2	3	0.013	0.908
No	59	156		
Drinking				
Yes	6	10	0.380	0.537
No	55	149		
Histologic grade				
G3	28	20	28.699	< 0.0005
G1/2	33	139		
Clinical stages				
III–IV	18	15	13.933	< 0.0005
I–II	43	144

**Table 4:** The association between lymph node metastasis and clinicopathological features (multivariate analysis).

	HR	95% CI	*P*
Tumor size (cm)	3.50	1.72–7.67	< 0.001
Ki-67	1.98	0.91–4.21	0.09
NLR	2.48	1.18–5.01	0.015
Histologic grade	0.98	0.41–2.50	0.99

## Discussion

In this study, we found that the preoperative NLR was significantly higher in patients with axillary lymph node metastasis than in those without lymph node metastasis. This study focused on the relationship between NLR and lymph node metastasis. We then investigated the predictive value of NLR in cN0 HR(+) breast cancer lymph node metastases. The optimal cutoff value for NLR was 2.4, which was nearly identical to that of the population of Japan^17^. Our results indicate that a preoperative elevated NLR is a significant factor for predicting lymph node metastasis in cN0 HR(+) breast cancer patients. It is useful to reduce the complications of unnecessary ALND. While other biomarkers such as tumor size and Ki-67 levels are commonly used to predict lymph node metastasis, NLR offers a non-invasive, cost-effective alternative with a robust predictive value for cN0 HR(+) breast cancer patients.

An increasing number of studies have recently revealed that the occurrence of cancers is linked to the inflammatory response. Tumor growth, invasion, angiogenesis, and ultimately metastasis can all be aided by inflammation^18^. The systemic inflammatory response can vary with tumor growth, invasion, and metastasis. As a result, various inflammation-based predictors are routinely used to predict the prognosis of various cancers. In clinical practice, the white blood cell (WBC) count is one of the most valuable inflammatory indicators. It is also a simple laboratory measure used to assess systemic inflammation. There has been much interest in the idea that the systemic inflammatory response, specifically the NLR, can predict survival in breast cancer patients. Azab et al.^19^ investigated 465 breast cancer patients and found that those with an increased NLR had a worse prognosis. Similar findings have been observed in other investigations^20,21^. Axillary lymph node metastases are a significant predictive marker and one of the most critical variables in determining treatment decisions in breast cancer patients. As a result, determining the extent of metastases in the axillary lymph nodes is vital for surgery, medical therapy, and prognosis^22^.

The gold standard for axillary staging in cT1-2N0M0 breast cancer patients is SLNB, and the preferred SLN tracer method is the dye mixed isotope double tracer approach. However, most Chinese hospitals still employ the single dye approach of methylene blue for tracing due to isotope application limitations. Even some hospitals are not equipped to perform SLNB. To avoid the complications of unneeded ALND, finding an efficient and less invasive biomarker indicator for predicting lymph node metastases is critical.

It is well known that tumor size plays an important role in predicting axillary lymph node status in patients with breast cancer^23^. According to Koh et al.^21^, a higher NLR was associated with a larger tumor, a higher histologic grade, lymph node metastases, and distant metastases. Likewise, we analyzed the association between NLR level and clinicopathological characteristics, and the results showed that patients with elevated NLR had a higher risk of lymph node metastases, larger tumors, and higher histologic grades. At the same time, we investigated the relationship between NLR and the clinical stage in our research. Thirty-three patients were in stage III/IV (33/220, 15%). One hundred eighteen patients with a low NLR (118/129, 91.5%) were in stage I/II (early stage) at diagnosis, and 11 patients (11/129, 8.5%) were in stage III/IV. In addition, 69 individuals in the high NLR category (69/91, 75.8%) were in the early stage, and 22 patients (22/91, 24.2%) were in stage III/IV. The NLR was found to have an association with the clinical stage after statistical analysis. The link between a high NLR and a high stage of the disease could be explained by lymphocytes' ability to slow the progression of cancer. Tumor-infiltrating lymphocytes have been proven to improve cancer patient survival rates^24^. As a result, a low number of lymphocytes resulted in a high NLR, which could imply a link between high stage and axillary lymph node metastases. According to Laohawiriyakamol et al.^25^, NLR is a valuable diagnostic tool for predicting additional non-SLN metastases. Our study results agree that NLR has predictive efficacy in the diagnosis of lymph node metastasis. The study by Modica^26^ and our own research both highlight the importance of inflammatory markers like NLR in cancer prognosis. Modica investigated the role of NLR in medullary thyroid cancer (MTC) and found it indicative of systemic inflammatory responses linked to tumor behavior and patient outcomes. Our findings similarly demonstrate a significant correlation between elevated preoperative NLR and axillary lymph node metastasis in cN0 HR(+) breast cancer patients, suggesting that systemic inflammation is crucial for cancer progression. These consistent results across multiple studies advocate for further investigation into inflammation-based predictors like NLR for improving cancer management and diagnostic accuracy. Based on the research conducted by Howard et al.^27^ it was shown that NLR has potential value as a prognostic biomarker across different cancer types and clinical stages. In this study, NLR was selected as the primary marker for its strong correlation with cancer prognosis, particularly in breast cancer, and has proven more effective than other markers like PLR and LMR.

However, there are of course some exceptions. According to Yang et al.^28^, although the SLN metastatic rate in the elevated NLR group was higher than that in the lower NLR group in univariate analysis, the difference was not statistically significant in multivariate analysis. This result suggests the complexity of inflammatory markers and their interactions with cancer stage and other prognostic factors. These differences might be due to our study group including a high proportion of patients with early-stage disease. Moreover, cN0 HR(+) breast cancer patients have a better prognosis. Of course, with a larger sample size, it is possible to acquire results with a significant difference. Previous studies have also highlighted the role of systemic inflammation markers like NLR in predicting cancer prognosis^19,21^. However, these studies primarily focused on varied cancers, and few have explored NLR specifically within the context of HR(+) breast cancer. This research gap is addressed in our study by examining the predictive efficacy of NLR specifically in cN0 HR(+) breast cancer. Our findings are supported by recent literature suggesting the inflammatory response's role in cancer progression, where a high NLR correlates with worse outcomes^26^. This study contributes incrementally to the understanding of NLR's role in breast cancer by confirming its predictive value for lymph node metastasis in a specific patient group—those with cN0 HR(+) status. It builds on previous findings by providing additional evidence of NLR's utility in reducing unnecessary axillary lymph node dissections and guiding treatment planning. Despite its retrospective nature, the study lays groundwork for prospective trials.

To the best of our knowledge, this is one of the few studies to look into the link between NLR and axillary lymph node metastases in cN0 HR(+) breast cancer. We found that the preoperative NLR in cN0 HR(+) breast cancer patients can be used as a predictor for lymph node metastasis. An obvious flaw of this study is that it is a retrospective analysis. As a result, larger-scale prospective trials with a larger sample size are needed in the future to confirm this finding and determine the cutoff value in different races. Despite this limitation, this investigation provides credible evidence for assessing the NLR impact in cN0 HR(+) breast cancer patients.

### Ethical approval

The Shaoxing Central Hospital's Institutional Review Committee approved this study, and the document approval number is 2023–27.

## Conclusion

The NLR is widely available information that can assist surgeons in making prompt judgments on ALND for their patients. The NLR, as an accessible, low-cost, and effective biomarker, may be added to current prognostic indicators in the future to determine treatment options for breast cancer patients.

## Data Availability

The datasets generated and analysed during the current study are not publicly available due do not have consent from all patients to publish this data, but are available from the corresponding author on reasonable request.

## References

[1] Sung H (2021). Global cancer statistics 2020: GLOBOCAN estimates of incidence and mortality worldwide for 36 cancers in 185 countries. CA Cancer J. Clin..

[2] Zeng H (2021). Disparities in stage at diagnosis for five common cancers in China: A multicentre, hospital-based, observational study. Lancet Public Health.

[3] Page DL, Jensen RA, Simpson JF (1998). Routinely available indicators of prognosis in breast cancer. Breast Cancer Res. Treat..

[4] Hanahan D, Weinberg RA (2011). Hallmarks of cancer: The next generation. Cell.

[5] Kolb, R. & Zhang, W. Obesity and breast cancer: A case of inflamed adipose tissue. *Cancers (Basel)*. **12**(6) (2020). 10.3390/cancers12061686 PMC7352736 32630445

[6] Coussens LM, Werb Z (2002). Inflammation and cancer. Nature.

[7] Greten FR, Grivennikov SI (2019). Inflammation and cancer: Triggers, mechanisms, and consequences. Immunity.

[8] Mantovani A (2009). Cancer: Inflaming metastasis. Nature.

[9] Haddad CR (2015). Neutrophil-to-lymphocyte ratio in head and neck cancer. J. Med. Imaging Radiat. Oncol..

[10] Kang MH (2014). The prognostic impact of the neutrophil-to-lymphocyte ratio in patients with small-cell lung cancer. Br. J. Cancer.

[11] Feng JF, Huang Y, Liu JS (2013). Combination of neutrophil lymphocyte ratio and platelet lymphocyte ratio is a useful predictor of postoperative survival in patients with esophageal squamous cell carcinoma. Onco Targets Ther..

[12] Xue P (2014). Neutrophil-to-lymphocyte ratio for predicting palliative chemotherapy outcomes in advanced pancreatic cancer patients. Cancer Med..

[13] Rachidi S (2016). Neutrophil-to-lymphocyte ratio and overall survival in all sites of head and neck squamous cell carcinoma. Head Neck.

[14] Amin MB (2016). AJCC Cancer Staging Manual.

[15] Muftah AA (2017). Ki67 expression in invasive breast cancer: the use of tissue microarrays compared with whole tissue sections. Breast Cancer Res. Treat..

[16] Wolff AC (2018). HER2 testing in breast cancer: American Society of Clinical Oncology/College of American Pathologists clinical practice guideline focused update summary. J. Oncol. Pract..

[17] Nakano K (2014). Prognostic significance of pre-treatment neutrophil: Lymphocyte ratio in Japanese patients with breast cancer. Anticancer Res..

[18] Lee JA (2011). D2–40, podoplanin, and CD31 as a prognostic predictor in invasive ductal carcinomas of the breast. J. Breast Cancer.

[19] Azab B (2012). Usefulness of the neutrophil-to-lymphocyte ratio in predicting short- and long-term mortality in breast cancer patients. Ann. Surg. Oncol..

[20] Dirican A (2015). Do the derived neutrophil to lymphocyte ratio and the neutrophil to lymphocyte ratio predict prognosis in breast cancer?. Int. J. Clin. Oncol..

[21] Koh CH (2015). Utility of pre-treatment neutrophil-lymphocyte ratio and platelet-lymphocyte ratio as prognostic factors in breast cancer. Br. J. Cancer.

[22] Giuliano AE (2017). Breast Cancer-Major changes in the American Joint Committee on Cancer eighth edition cancer staging manual. CA Cancer J. Clin..

[23] Schwartz RS, Erban JK (2017). Timing of metastasis in breast cancer. N. Engl. J. Med..

[24] Ohashi R (2006). Prognostic factors in patients with inoperable non-small cell lung cancer–an analysis of long-term survival patients. Gan To Kagaku Ryoho.

[25] Laohawiriyakamol S (2017). The pre-treatment neutrophil-lymphocyte ratio: A useful tool in predicting non-sentinel lymph node metastasis in breast cancer cases. Asian Pac. J. Cancer Prev..

[26] Modica R (2023). Evaluation of Neutrophil-to-Lymphocyte Ratio (NLR), Platelet-to-Lymphocyte Ratio (PLR) and Systemic Immune-Inflammation Index (SII) as Potential Biomarkers in Patients with Sporadic Medullary Thyroid Cancer (MTC). J. Pers. Med..

[27] Howard R (2019). Exploring the prognostic value of the neutrophil-to-lymphocyte ratio in cancer. Sci. Rep..

[28] Yang L (2021). Association between the platelet to lymphocyte ratio, neutrophil to lymphocyte ratio and axillary lymph node metastasis in cT1N0 breast cancer patients. Am. J. Transl. Res..

